# Enface vitreous OCT ‘worm holes’: A novel finding in a patient with diffuse unilateral subacute neuroretinitis (DUSN)

**DOI:** 10.1016/j.ajoc.2021.101112

**Published:** 2021-05-12

**Authors:** Simrat K. Sodhi, John Golding, Efrem D. Mandelcorn, Andrea K. Boggild, Netan Choudhry

**Affiliations:** a University of Cambridge, The Old Schools, Trinity Lane, Cambridge, UK; b Vitreous Retina Macula Specialists of Toronto, 3280 Bloor St. West, Suite 310, Etobicoke, ON, Canada; c The Donald K. Johnson Eye Centre, Department of Ophthalmology and Vision Sciences, Toronto Western Hospital, University Health Network, University of Toronto, 399 Bathurst St, Toronto, ON, Canada; d Tropical Disease Unit, Toronto General Hospital, Canada; e Division of Infectious Diseases, University Health Network, Canada; f Department of Ophthalmology and Vision Sciences, University of Toronto, 340 College Street, Suite 400, Toronto, ON, Canada; g Cleveland Clinic Canada, 181 Bay Street, Brookfield Place, 30th Floor, Toronto, ON, Canada

**Keywords:** Diffuse unilateral subacute neuroretinitis, Multimodal imaging, Multicolor scanning laser imaging, Enface swept source vitreous optical coherence tomography, Nematode

## Abstract

**Purpose:**

To describe a case of diffuse unilateral subacute neuroretinitis (DUSN), a rare condition that causes progressive vision loss following infection by a nematode using enface vitreous imaging.

**Observations:**

We present the clinical findings of a 37-year-old female, clinically diagnosed with DUSN after a non-invasive multimodal imaging approach that included MultiColor scanning laser imaging and enface vitreous OCT, which revealed the nematode body and lacunae created by worm migration, respectively.

**Conclusion and importance:**

To our knowledge, this is the first reported case of lacunae visualized using enface vitreous optical coherence tomography (OCT), potentially marking the migration path of the nematode.

## 1 Introduction

Diffuse unilateral subacute neuroretinitis (DUSN) is a rare, ocular infectious disease caused by infection by a nematode that can lead to severe visual impairment and blindness. It primarily presents unilaterally, however bilateral cases have been previously reported.^1^ DUSN typically occurs in young, otherwise healthy, individuals, a finding Gass noted when he first described the disease in 1977.^2^

Previously termed unilateral wipeout syndrome, DUSN presents with: “(1) insidious, usually severe loss of peripheral and central vision; (2) vitritis; (3) diffuse and focal pigment epithelial derangement with relative sparing of the macula; (4) narrowing of the retinal vessels; (5) optic atrophy; (6) increased retinal circulation time; (7) subnormal electroretinographic findings.”^2^ Given the aforementioned clinical features, DUSN can often masquerade as optic neuritis, pars planitis, histoplasmosis, toxoplasmosis, syphilitic chorioretinitis, sarcoidosis, acute posterior multifocal placoid pigment epitheliopathy, choroidal macrovessel and multiple evanescent white dot syndrome (MEWDS) in the early stages as well as unilateral retinitis pigmentosa, occlusive vascular disease, toxic retinopathy, and posttraumatic chorioretinopathy in the late stages.^3^

In 60–75% of cases, the nematode is not visualized and thus surgical or laser intervention is not possible.^4^ In patients where the worm is not observed, treatment with oral anthelmintic drugs, such as albendazole, can be used, but according to previous reports only some of the subretinal worms were destroyed due to differences in the blood-retinal barrier breakdown of the inflamed eye.^3^ We describe a case of DUSN evaluated using multi-modal imaging, where the nematode was visible, following MultiColor scanning laser imaging, on the internal limiting membrane (ILM). Enface vitreous optical coherence tomography (OCT) further revealed lacunae, which may represent areas of worm movement (“worm holes”). To our knowledge this has not been previously reported and may aid in the visualization of nematodes in suspected DUSN cases.

## 2 Case report

A 37-year-old female with sudden vision loss in the left eye was referred for possible MEWDS. The patient works as a social media manager and had no past medical or ocular history that was pertinent to this case. There was no family history of any ocular or genetic diseases. In November 2019, the patient travelled to Lahore, Pakistan where she consumed street food that contained beef, chicken and fish. The meat was either grilled or fried and no uncooked or raw meat was consumed. The patient returned home to Toronto in January 2020, asymptomatic from her travel. In May 2020, the patient was gardening and handled racoon feces, while wearing disposable gloves. Within one month, the patient noticed sudden decreased vision in the left eye. Her best-corrected visual acuity (BCVA) was 20/20 OD and CF OS. Anterior segment examination was unremarkable. Afferent pupillary defect was noted OS. The right eye was normal. Fundus examination revealed the presence of 1+ vitreous cells in the left eye with a normal appearing disc, the macular and posterior pole demonstrated diffuse non-specific retinal pigment epithelium (RPE) changes and normal appearing retinal vessels. OCT revealed nerve fibre layer (NFL) thinning in the affected eye. No obvious change in the choroid was noted.

**Fig. 1:**
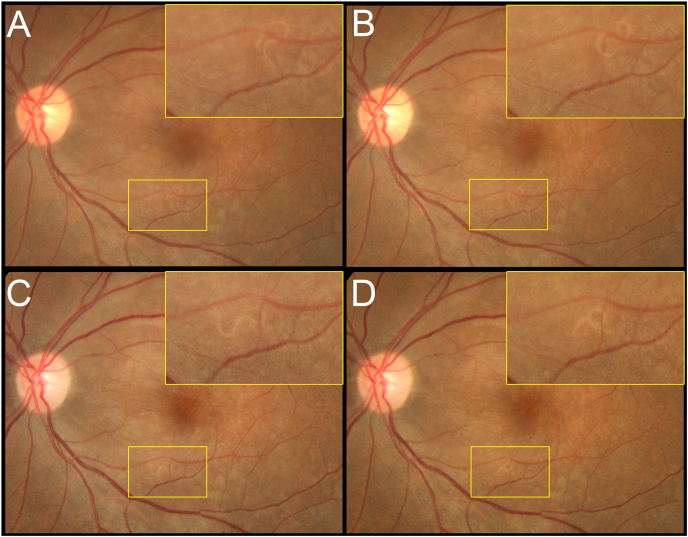
(A–D) Color fundus photographs (Topcon TRC 50-DX; Tokyo, Japan) of the left eye demonstrating the subretinal nematode in motion. Magnified inset images corresponds to area demarcated in yellow. (For interpretation of the references to color in this figure legend, the reader is referred to the Web version of this article.)

**Fig. 2:**
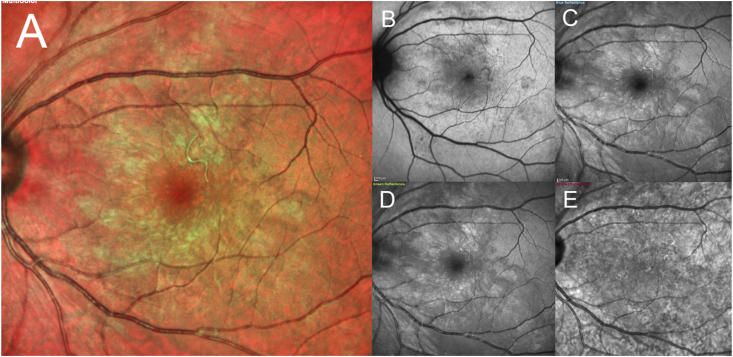
MultiColor scanning laser imaging (Heidelberg Spectralis HRA + OCT MultiColor; Heidelberg Engineering, Inc., Heidelberg, Germany) of the left eye demonstrating nematode located on the retinal surface. **(A)** Multicolor composite image; **(B)** Blue light autofluorescence; **(C)** Blue reflectance image; **(D)** Green reflectance image; **(E)** Near-infrared reflectance image. (For interpretation of the references to color in this figure legend, the reader is referred to the Web version of this article.)

**Fig. 3:**
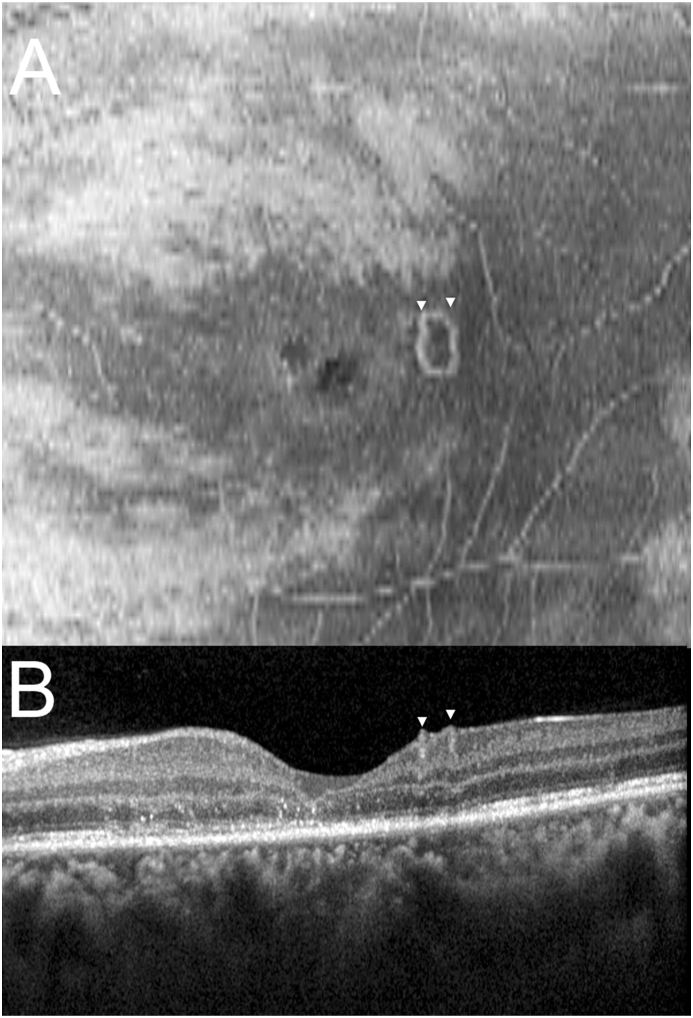
**(A)** Enface OCT (Heidelberg Spectralis) segmented at the ILM demonstrating the nematode (arrowheads) atop the ILM in a coiled position; **(B)** Corresponding transverse SD-OCT image.

Initially, color fundus photography and OCT was performed, but the nematode was not visible. Fundus autofluorescence (FAF) (Optos California; Optos PLC; Dunfirmline, UK) demonstrated both focal and pinpoint hyper-and hypo-autofluorescent dots in the posterior pole and along the arcade, but did not show a nematode body. MultiColor scanning laser imaging (Heidelberg Spectralis HRA + OCT MultiColor; Heidelberg Engineering, Inc., Heidelberg, Germany) revealed the nematode body (Fig. 2A), which was observed in the green reflectance (GR) and blue reflectance (BR) images (Fig. 2C&D), while near-infrared reflectance (NIR) did not show the worm (Fig. 2E). Subsequent color fundus photography (Topcon TRC 50-DX) revealed a subretinal nematode in the inferotemporal quadrant of the left eye (Fig. 1A–D). Enface OCT (Heidelberg Spectralis HRA + OCT) segmented at the ILM demonstrated the nematode atop the ILM in a coiled position (Fig. 3A), which is incompletely visible in the corresponding transverse SD-OCT images (Fig. 3B). Enface swept-source OCT of the vitreous (Plex Elite 9000; Carl Zeiss Meditec, Germany) revealed hyper-reflective dots suggestive of vitreous cells and several opaque small lacunae temporally and inferotemporally suggestive of, what we would term, “worm holes” (Fig. 5). These worm holes are potentially areas where the nematode traversed through the vitreous.

**Fig. 4:**
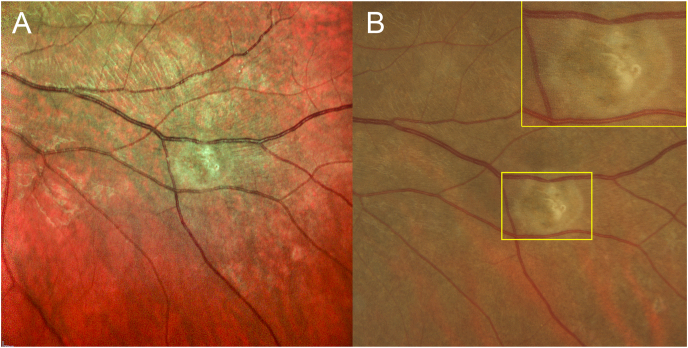
**(A)** MultiColor scanning laser imaging and **(B)** color fundus photograph of the left eye post-laser treatment demonstrating early laser burn with centrally locating nematode corpse. Magnified inset corresponds to area demarcated in yellow. (For interpretation of the references to color in this figure legend, the reader is referred to the Web version of this article.)

After her initial diagnosis of presumed MEWDS, further serological testing (i.e. rapid plasma reagin, PPD skin test) was initiated, but halted once the nematode body was discovered. The patient subsequently received laser photocoagulation (power = 200 mW; spot size = 100 μ; lens area centralis duration = 0.2 ms; 41 spots) in a circinate ring around the nematode with central spots delivered directly on the worm body. After treatment, the patient remained CF OS, but in post-laser imaging at one week, the worm carcass was visible in the inferotemporal quadrant of the fundus (Fig. 4A&B). One month after the laser procedure, the patient's vision improved to 20/200 OS.

**Fig. 5:**
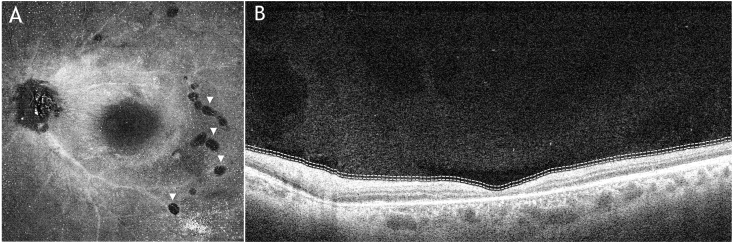
**(A)** Enface swept-source vitreous OCT approximately 10 μm above the internal limiting memebrane (ILM) (Plex Elite 9000; Carl Zeiss Meditec, Germany) of the left eye demonstrating hyperreflective dots suggestive of cells and several small hypo-reflective lacunae (arrowheads) temporally and inferotemporally suggestive of worm holes from previous worm movement; **(B)** Corresponding b-scan image.

## 3 Discussion

DUSN is an ocular infection caused by several nematodes, including *Toxocara canis*, *Baylisascaris procyonis* and larvae of *Ancylostoma caninum*.^3^ The worm may be introduced if nematode eggs are ingested or inhaled. The length of the worm was measured after MultiColor scanning laser imaging (Heidelberg Spectralis) using the built-in calipers and was approximately 1180 μm. The worm was not surgically removed and stool examination was not performed; therefore we were unable to confirm the worm strain; however, given the handling of racoon feces and size of the worm, we propose *B. procyonis* to be the etiological agent.^3^ The geographical location provides further evidence of infection with *B. procyonis*, as larger worms (1500–2000 μm in length) are associated with northern midwestern United States. The majority of the western hemisphere is associated with smaller worms (400–700 μm in length), which makes them harder to visualize.^5^^,^^6^ Guidance on systemic treatment and investigation was obtained from infectious disease consultation.

*B. procyonis* rapidly moves in and out of the retina, adding to the difficulty in capturing its image. It exhibits random movement through all the retinal layers, not only the subretinal space and thus causes vast damage to the retina while minimally altering the choroid.^7^ However, there may be innate nematode behaviours that can be exploited to impact movement. For example, Nematodes, particularly *Caenorhabditis elegans*, are used in real-time survival bioassays that utilize blue LED light (450–490nm) to evaluate their survival. In these studies, nematodes that stopped moving under white light (360–760nm) responded rapidly to blue light.^8^ Blue light is considered a noxious stimulus and thus nematodes tend to exhibit classic avoidance behaviors during exposure. A classic “omega turn” pattern was seen in color fundus photographs taken after blue light stimulation (Fig. 1A).^9^ *B. procyonis* may exhibit similar properties to *C. elegans*, but this is purely speculation and would need to be further evaluated in future studies.

As part of the imaging workup, we used the Heidelberg Spectralis HRA + OCT MultiColor system. MultiColor scanning laser imaging simultaneously scans 3 individual laser wavelengths, including blue light: 820 nm (NIR), 515 nm (GR) and 488 nm (BR).^10^ Prior to its use, the nematode was not visible, potentially caused by its location out of the field of view or in the subretinal space. However, stimulation by the blue laser might have been associated with the worm becoming motile. Other blue light modalities, such as blue-light fundus autofluorescence (BAF) (Heidelberg Spectralis), may illicit a similar response as described above because it uses a 488 nm excitation laser; however, we are unable to comment on this as our initial FAF imaging used the Topcon fundus camera, which employs a 535–580 nm excitation ﬁlter and a 615–715 nm barrier instead of a blue light laser (Fig. 2B).^11^ However, this hypothesis is entirely speculative and would require further clinical and basic research to elucidate the basis of nematode motility.

Enface OCT scans, provides a high resolution, confocal OCT image of the macular area combined with a transverse image of the same area.^12^ This approach allowed visualization of the nematode that was otherwise difficult to see in the transverse OCT image. In cases where the nematode is not visible or the OCT shows anatomical features consistent with DUSN, enface OCT may allow for clear visualization of the worm body. Enface vitreous OCT (Fig. 5A) also revealed hyporeflective, well circumscribed areas which we suggest may represent lacunae in the vitreous formed by worm movement in the temporal and inferotemporal vitreous over the posterior pole. This is suggested by their smaller size compared to age-related lacunae and their location just above where the worm was identified.^13^ Fig. 5B shows the corresponding b-scan, where these lacunae can be seen as dark voids in the vitreous. The nematode may have moved in and out of the vitreous and retina before embedding itself in the subretinal space.

Following laser photocoagulation, the carcass of the nematode remained visible in the inferotemporal quadrant of the left eye (Fig. 4A&B). Natesh et al., reported a similar finding in their 2010 case report in which the nematode remained active post-laser treatment.^14^ In their report, the nematode was determined to be alive when it moved away from the lasered area and was killed with subsequent laser treatment. In our case, the nematode carcass was present within the lasered area, but to definitively confirm the nematode was dead, we imaged the area several times, using MultiColor (Fig. 4A), and each time the nematode was in a consistent, crisped position.

## 4 Conclusion

This is the first report to show nematode visualization using a full non-invasive multimodal approach. Each of the imaging techniques involved revealed anatomic patterns that were pertinent to the case. The nematode was visualized during the image capture process; therefore, it may be worthwhile for ophthalmologists to use a multimodal approach that includes modalities that utilize a blue light laser to potentially stimulate the nematode enough to trigger motility. Enface vitreous OCT was also utilized to visualize lacunae and may provide an additional tool in assisting in the diagnosis of DUSN when the nematode body is not found. These lacunae should not be confused with age related lacunae, which occur in older patients.

## Patient consent

Consent to publish this case report has been obtained from the patient in writing.

## Funding

No funding or grant support.

## Authorship

All authors attest that they meet the current ICMJE criteria for authorship.

## Acknowledgments

None.

## Declaration of competing interest

Netan Choudhry is a consultant and receives research equipment from Topcon, Optos and Carl Zeiss Meditec. None of the aforementioned companies had a role in the study design, collection, analysis and interpretation of data in this report. The following authors have no financial disclosures: SKS, JG, EDM.
